# Associations between psychological therapy outcomes for depression and incidence of dementia

**DOI:** 10.1017/S0033291722002537

**Published:** 2023-08

**Authors:** Amber John, Rob Saunders, Roopal Desai, Georgia Bell, Caroline Fearn, Joshua E. J. Buckman, Barbara Brown, Shirley Nurock, Stewart Michael, Paul Ware, Natalie L. Marchant, Elisa Aguirre, Miguel Rio, Claudia Cooper, Stephen Pilling, Marcus Richards, Josh Stott

**Affiliations:** 1 ADAPT Lab, Clinical, Educational and Health Psychology, UCL, London; 2 Research Department of Clinical, Centre for Outcomes and Research Effectiveness, Educational and Health Psychology, UCL, London, UK; 3 iCope – Camden and Islington Psychological Therapies Services, Camden & Islington NHS Foundation Trust, London, UK; 4 Division of Psychiatry, UCL, London, UK; 5 North East London NHS Foundation Trust (NELFT), London, UK; 6 Department of Electronic and Electrical Engineering, UCL, London, UK; 7 Centre for Psychiatry and Mental Health, Queen Mary University of London, London, UK; 8 Camden & Islington NHS Foundation Trust, St Pancras Hospital, London, UK; 9 MRC Unit for Lifelong Health and Ageing at UCL, London, UK

**Keywords:** Dementia, depression, psychological therapy, psychological treatment, treatment outcome

## Abstract

**Background:**

Depression is an important, potentially modifiable dementia risk factor. However, it is not known whether effective treatment of depression through psychological therapies is associated with reduced dementia incidence. The aim of this study was to investigate associations between reduction in depressive symptoms following psychological therapy and the subsequent incidence of dementia.

**Methods:**

National psychological therapy data were linked with hospital records of dementia diagnosis for 119808 people aged 65+. Participants received a course of psychological therapy treatment in Improving Access to Psychological Therapies (IAPT) services between 2012 and 2019. Cox proportional hazards models were run to test associations between improvement in depression following psychological therapy and incidence of dementia diagnosis up to eight years later.

**Results:**

Improvements in depression following treatment were associated with reduced rates of dementia diagnosis up to 8 years later (HR = 0.88, 95% CI 0.83–0.94), after adjustment for key covariates. Strongest effects were observed for vascular dementia (HR = 0.86, 95% CI 0.77–0.97) compared with Alzheimer's disease (HR = 0.91, 95% CI 0.83–1.00).

**Conclusions:**

Reliable improvement in depression across psychological therapy was associated with reduced incidence of future dementia. Results are consistent with at least two possibilities. Firstly, psychological interventions to improve symptoms of depression may have the potential to contribute to dementia risk reduction efforts. Secondly, psychological therapies may be less effective in people with underlying dementia pathology or they may be more likely to drop out of therapy (reverse causality). Tackling the under-representation of older people in psychological therapies and optimizing therapy outcomes is an important goal for future research.

## Introduction

Prevention is a global healthcare priority for dementia care and research (Livingston et al., 2020). Approximately 40% of dementia cases are associated with a range of potentially modifiable risk factors, including depression in late life (Livingston et al., 2020). Systematic reviews and meta-analyses have shown that depression is significantly associated with increased risk of all-cause dementia (Da Silva, Gonçalves-Pereira, Xavier, & Mukaetova-Ladinska, 2013; Jorm, 2001), Alzheimer's disease (Diniz, Butters, Albert, Dew, & Reynolds, 2013; Ownby, Crocco, Acevedo, John, & Loewenstein, 2006), and vascular dementia (Diniz et al., 2013), with evidence showing that the association between depression and vascular dementia is stronger than with other dementia outcomes (Barnes et al., 2012; Diniz et al., 2013). There are multiple potential mechanisms which may underlie observed associations between depression and dementia. Specifically, depression may (1) act as an etiological factor for dementia; (2) be a prodromal feature of dementia; (3) share a common genetic or neurophysiological cause; or (4) may be a response to emerging cognitive impairment (reverse causality) (Butters et al., 2008).

Although there is evidence that depression is associated with dementia, it is currently unclear whether successful treatment of depressive symptoms is associated with reduced incidence of dementia. Antidepressant medications [including selective serotonin reuptake inhibitors (SSRIs), tricyclics, serotonin and norepinephrine reuptake inhibitors (SNRIs), and monoamine oxidase inhibitors (MAOIs)] are widely used treatments for depression. It has been reported that older people are six times more likely to take medications for depression than their younger counterparts (Burns, 2015), though antidepressant use in older people has been associated with limited benefit and more adverse events (Mallery et al., 2019; Tham et al., 2016).

Psychological therapy such as cognitive behavioral therapy (CBT) is an effective first-line treatment for depression (National Institute for Health and Clinical Excellence (NICE), 2009). Patients frequently indicate preferences for psychological over pharmacological interventions (McHugh, Whitton, Peckham, Welge, & Otto, 2013), and these therapies may provide longer-term relapse prevention benefits (Clarke, Mayo-Wilson, Kenny, & Pilling, 2015; Cuijpers et al., 2013).

The aim of this study was to investigate associations between reduction in depressive symptoms following psychological therapy and the incidence of dementia diagnosis among adults aged 65 and over at the time of their treatment. Given that there are differences in the strength of associations between depression and different types of dementia with a particularly strong relationship with vascular dementia, the secondary aim was to test the associations between improvement in depression following psychological therapy and different sub-types of dementia diagnosis (Alzheimer's disease or vascular dementia).

## Methods

### Sample

The sample was drawn from individuals who accessed any Improving Access to Psychological Therapies (IAPT) services with clinically significant levels of depression from 2012 to 2019 in England. IAPT services operate in every area of England and provide evidence-based psychological therapy for common mental disorders (CMDs) such as depression and anxiety disorders, using a stepped-care model of delivery (Clark, 2018). All IAPT services collect the same standardized minimum dataset (the IAPT MDS) (National Collaborating Centre for Mental Health, 2021) of patient and treatment variables, including outcome measures and treatment information at each contact with the service. All IAPT services contribute to the national IAPT dataset (NHS Digital, 2020b), which contains detailed outcome data for over four million people that entered treatment since 2012. Scores of depression and anxiety pre-treatment and post-treatment are available on 98.5% of all patients who have at least two sessions before discharge (Clark, 2018).

Data for people who accessed IAPT services up to 2019 were linked with other national healthcare data, including Hospital Episode Statistics (HES) up to 2020 (NHS Digital, 2021a) (Admitted Patient Care and Outpatient datasets), HES-ONS linked mortality data up to 2020 (NHS Digital, 2020a), and the Mental Health Services Dataset (MHSDS) up to 2019 (NHS Digital, 2021b) using a linkage key provided by NHS Digital (online Supplementary Materials S1). Of the sample eligible for this study, only a small proportion of patients had no linked data in HES, MHMDS/MHLDDS/MHSDS, or ONS death record. Of 122 999 people meeting the above criteria, only 1761 (1.43%) had no linked record in any other dataset.

### Participants

The analytic sample was comprised of all individuals aged 65 or above at baseline in the linked dataset who accessed psychological therapies (including both low and high intensity) and received a course of treatment [defined as 2 or more treatment sessions by national reports (NHS Digital, 2020b). Any individuals with a dementia diagnosis, identified in the HES or MHSDS datasets, prior to entering IAPT treatment were excluded from analyses (*N* = 2437). Additionally, individuals who scored below the clinical threshold for caseness depression (⩽9 on PHQ9) (Kroenke, Spitzer, & Williams, 2001) were also excluded from the analytic sample. Where individuals accessed psychological therapy services on more than one occasion over the study period the first recorded episode was used, to maximize the length of follow up available. Participants were also excluded where the last recorded contact with health services was prior to their psychological therapy. It is possible that undiagnosed dementia may be present during therapy. In order to try and ensure these cases did not exert a disproportionate effect on results, participants who had dementia diagnosed within 1 year of ending psychological therapy were excluded.

### Measures

#### Main exposure

Depressive symptoms were measured using the 9-item Patient Health Questionnaire (PHQ-9, range 0–27) (Kroenke et al., 2001). The main exposure measure was reliable improvement in depression, defined as a reduction of ⩾6 points on the PHQ-9. For online Supplementary analyses, models were re-run using reliable recovery from depression as an effect. Reliable recovery is defined, as both: (1) showing reliable improvement in depression (as defined above), and (2) moving from a score above the clinical threshold for case-level depression (⩾10 points on PHQ-9) before therapy to below the clinical threshold for depression at the final treatment appointment. Cut-offs were used based on national IAPT guidelines (National Collaborating Centre for Mental Health, 2021). Online Supplementary analyses also used a continuous change score of depression symptoms across therapy. This was derived by calculating the difference between the PHQ-9 score at entry to the service and at discharge from the service.

#### Main outcome

Based on previously published procedures (Singh-Manoux et al., 2017), ICD-10 codes were used to identify all-cause dementia, Alzheimer's disease and vascular dementia cases in HES, MHSDS and mortality datasets. Previous research has shown that there is a high agreement rate in dementia case identification between HES and GP records (Brown et al., 2016). The primary outcome was all-cause dementia, and additional analyses were run, using the two commonest forms of dementia (Alzheimer's disease and vascular dementia) as the outcome.

#### Covariates

Covariates were selected based on known associations with psychological therapy outcomes, and dementia risk (Buckman et al., 2021; Saunders, Buckman, & Pilling, 2020). Demographic covariates were age, gender, ethnicity (based on UK census codes), and deprivation [measured using the Index of Multiple Deprivation (IMD)]. Clinical covariates were baseline depression severity, number of attended therapy sessions, comorbid anxiety (⩾8 on GAD-7) (Spitzer, Kroenke, Williams, & Löwe, 2006), whether the cardiovascular disease was ever recorded, self-reported presence of long-term health condition, and whether patients reported taking psychotropic medication at the time of therapy. Patients who were prescribed psychotropic medication but reported not taking these [*N* = 5126, (4.8%)] were coded as ‘not taking’. All demographic and clinical covariates were available in the IAPT dataset at the time of initial assessment, apart from cardiovascular disease diagnosis, which was available in HES data.

### Statistical analysis

#### Main analyses

The sample with complete information on all key variables and covariates was compared with the sample with missing data in order to test whether they differed on key variables and covariates. Comparisons were made using *t* tests and χ^2^ tests as appropriate.

Next, Cox proportional hazards models were used to test associations between reliable improvement in depression following psychological therapy, and incident dementia. Time to event was measured in days from the end of psychological therapy. Participants were censored at the date of death, or at the participant's final recorded contact with services. The assumption of proportional hazards was checked using Schoenfeld residuals. Three main models were run. Model 1: Unadjusted; Model 2: Adjusted for demographic covariates only (age, gender, ethnicity, deprivation); Model 3: Adjusted for demographic and clinical covariates (psychotropic medications, number of attended therapy sessions, comorbid anxiety, cardiovascular disease, baseline depression severity, and long-term health conditions). For covariates with >5% missing (ethnicity, psychotropic medication use, and long-term health conditions), missingness was coded as a separate category so cases with missing covariate data could be included in main models and not subject to list-wise deletion (Saunders et al., 2021). This allowed the sample size to be maximized. All statistical analyses were conducted in Stata 16.

To test whether associations between reliable improvement in depression and incident dementia differ depending on the type of dementia diagnosis, models were re-run using the two most common forms of dementia (Alzheimer's disease and vascular dementia) as the primary outcomes. These analyses were each run excluding patients diagnosed with other types of dementia.

#### Supplementary analyses

First, planned online Supplementary analyses were conducted to test whether other measures of improvement from depression are associated with dementia incidence. Specifically, models were re-run using reliable recovery from depression and also using the continuous PHQ-9 change score across therapy as the main predictor. Next, although patients who had dementia diagnosed within 1 year of psychological therapy were excluded to minimize the effects of any undiagnosed dementia present during therapy, it is still possible that some patients may have undiagnosed dementia which is not diagnosed within the first year. Online Supplementary analyses were therefore run excluding patients who had dementia diagnosed within 2 years of ending psychological therapy. An extra online Supplementary analysis was also conducted stratifying the model by the median follow-up time (time to dementia, death, or last diagnosis). Another online Supplementary analysis was conducted including only the subgroup with moderate or severe depressive symptoms at the start of treatment (⩾15 points on the PHQ-9). Next, additional analyses were conducted excluding people who reported taking psychotropic medications at the time of therapy. Next, an analysis was run excluding people who showed reliable deterioration in anxiety over therapy. An additional model was run using all-cause mortality as an event and dementia diagnosis as censored observations. Finally, mixed effects Weibull survival models were conducted, including the IAPT service that offered therapy as a random effect, in order to test whether results remained consistent after accounting for a potentially nested data structure (i.e. patients within services).

## Results

### Descriptive statistics and missing data

The final analytic sample comprised 119 808 people. In total, 5340 (4.46%) patients had a dementia diagnosis recorded 1 year or more after ending psychological therapy. Of those with a dementia record, the mean time to diagnosis was 3.11 years (s.d. = 1.49). Of those without a dementia record, the mean length of follow up available was 3.28 years (s.d. = 1.87). Demographic characteristics for this sample are presented in Table 1. Demographic characteristics were also compared between the sample who were not diagnosed with dementia and the sample who were diagnosed with dementia at least 1 year after therapy (Table 2). Missing data analyses showed that in comparison to the sample with complete information for all key variables/covariates (*N* = 73 199; 61.1% of the sample), those with missing data (*N* = 46 609; 38.9% of the sample) were more likely to be female, older, of ethnic minority background, live in areas with higher levels of deprivation, and were less likely to show improvements in depression across therapy. Additionally, they were more likely to have a diagnosis of cardiovascular disease, have lower baseline depression severity, and were less likely to have comorbid anxiety (online Supplementary Table S1). Variables with >5% missing (ethnicity, psychotropic medication use, and long-term health conditions) were coded as a separate category, meaning that the sample size was maximized, as missing data was not subject to list-wise deletion.

**Table 1.** Demographic information for main analytic sample (*N* = 119 808)

	Missing *N* (%)	*N* (%)	Mean (s.d.)
Reliable improvement in depression	13 739 (11.47)		
No	–	37 082 (34.96)	–
Yes	–	68 987 (65.04)	–
Reliable recovery in depression	13 896 (11.60)		
No	–	44 654 (42.16)	–
Yes	–	61 258 (57.84)	–
Continuous change in depression score	415 (0.35)	–	7.20 (6.80)
Gender	452 (0.38)		
Male	–	38 271 (32.06)	–
Female	–	81 085 (67.94)	–
Age	0 (0.00)	–	71.55 (5.71)
Ethnicity	11 383 (9.50)		
White	–	103 737 (95.68)	–
Mixed	–	577 (0.53)	–
Asian	–	2330 (2.15)	–
Black	–	1022 (0.94)	–
Chinese	–	86 (0.08)	–
Other	–	673 (0.62)	–
IMD Decile	3999 (3.34)	–	5.91 (2.77)
Number of therapy sessions attended	0 (0.00)	–	6.23 (4.12)
Psychotropic medications	13 089 (10.92)		
No	–	46 989 (44.03)	–
Yes	–	59 730 (55.97)	–
Cardiovascular disease	93 (0.08)		
No	–	68 315 (57.06)	–
Yes	–	51 400 (42.94)	–
Comorbid anxiety	366 (0.31)		
No	–	16 035 (13.42)	–
Yes	–	103 407 (86.58)	–
Baseline depression severity	304 (0.25)	–	15.76 (4.59)
Long-term health condition	27 025 (22.56)		
No	–	41 302 (44.51)	–
Yes	–	51 481 (55.49)	–
Dementia diagnosis (from 1 year after therapy)	0 (0.00)		
No	–	114 468 (95.54)	–
Yes	–	5340 (4.46)	–
Time to dementia diagnosis (years)	0 (0.00)	–	3.11 (1.49)

**Table 2.** Comparison between sample who were not diagnosed with dementia (*N* = 114 468) and sample who were diagnosed with dementia at least 1 year after therapy (*N* = 5340)

	No Dementia	Dementia (at least 1 year after therapy)	*T* test/χ^2^
	(*N* = 114 468)	(*N* = 5340)	
Reliable improvement in depression: *N* (%)			
No	35 241 (34.74)	1841 (39.87)	χ^2^(1) = 51.27, *p* < 0.001
Yes	66 211 (65.26)	2776 (60.13)	
Reliable recovery in depression: *N* (%)			
No	42 465 (41.92)	2189 (47.51)	χ^2^(1) = 56.60, *p* < 0.001
Yes	58 840 (58.08)	2418 (52.49)	
Continuous change in depression score: Mean (s.d.)	7.25 (6.79)	6.18 (6.93)	*t*(119 391) = 11.22, *p* < 0.001
Gender: *N* (%)			
Male	36 494 (32.00)	1777 (33.40)	χ^2^(1) = 4.53, *p* = 0.03
Female	77 541 (68.00)	3544 (66.60)	
Age: Mean (s.d.)	71.36 (5.60)	75.65 (6.43)	*t*(119 806) = −54.39, *p* < 0.001
Ethnicity: *N* (%)			
White	99 196 (95.66)	4541 (96.04)	χ^2^(5) = 16.83, *p* = 0.005
Mixed	555 (0.54)	22 (0.47)	
Asian	2252 (2.17)	78 (1.65)	
Black	959 (0.92)	63 (1.33)	
Chinese	83 (0.08)	3 (0.06)	
Other	652 (0.63)	21 (0.44)	
IMD Decile: Mean (s.d.)	5.91 (2.77)	5.86 (2.81)	*t*(115 807) = 1.24, *p* = 0.22
Number of therapy sessions attended: Mean (s.d.)	6.26 (4.14)	5.58 (3.65)	*t*(119 806) = 11.75, *p* < 0.001
Psychotropic medications: *N* (%)			
No	45 117 (44.16)	1872 (41.13)	χ^2^(1) = 16.19, *p* < 0.001
Yes	57 051 (55.84)	2679 (58.87)	
Cardiovascular disease: *N* (%)			
No	66 653 (58.27)	1662 (31.16)	χ^2^(1) = 1500, *p* < 0.001
Yes	47 728 (41.73)	3672 (68.84)	
Comorbid anxiety: *N* (%)			
No	15 138 (13.26)	897 (16.89)	χ^2^(1) = 57.40, *p* < 0.001
Yes	98 993 (86.74)	4414 (83.11)	
Baseline depression severity: Mean (s.d.)	15.77 (4.58)	15.42 (4.70)	*t*(119 502) = 5.58, *p* < 0.001
Long-term health condition: *N* (%)			
No	39 664 (44.56)	1638 (43.37)	χ^2^(1) = 2.10, *p* = 0.15
Yes	49 342 (55.44)	2139 (56.63)	

### Psychological therapy outcomes for depression and incidence of dementia

In total 66 211 (65.26%) of people who did not develop dementia up to 8 years after therapy showed reliable improvement in symptoms of depression after psychological therapy. By comparison, of people who went on to develop dementia up to 8 years after therapy, only 2776 (60.13%) showed reliable improvement in depression after therapy.

Of people who did not reliably improve in symptoms of depression after therapy, 1841 (4.96%) went on to develop dementia up to 8 years later. Comparatively, of people who did reliably improve symptoms of depression, 2776 (4.02%) developed dementia up to 8 years later.

**Fig. 1.**
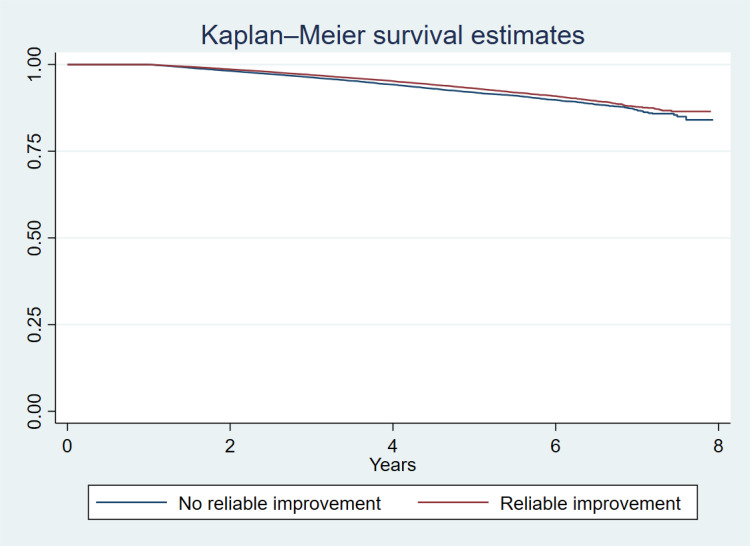
Kaplan Meier plot.

The Kaplan Meier curve is presented in Fig. 1. A Cox proportional hazards model showed that reliable improvement in depression across psychological therapy was associated with a 12% reduced rate of dementia diagnosis (HR = 0.88, 95% CI 0.83–0.94, *p* < 0.001) after full adjustment for all covariates (Table 3). The model also revealed that taking psychotropic medications was associated with increased rate of future dementia (HR = 1.17, 95% CI 1.09–1.24, *p* *<* *0*.001) (Table 3). Additionally, a greater number of attended sessions in IAPT was significantly associated with reduced rate of future dementia, though the effect size is small (HR = 0.98, 95% CI 0.98–0.99, *p* < 0.001). As expected, higher age was significantly associated with dementia incidence (HR = 1.11, 95% CI 1.11–1.12, *p* < 0.001). Baseline levels of depressive symptoms were not significantly associated with dementia incidence (HR = 1.00, 95% CI 0.998–1.01).

**Table 3.** Cox proportional hazards models to test associations between reliable improvement from depression and dementia incidence

	Model 1 (Unadjusted)	Model 2 (Adjusted for demographic covariates)	Model 3 (Adjusted for demographic and clinical covariates)^a^
Reliable improvement from depression	0.85 (0.80–0.90), <0.001^b^	0.86 (0.81–0.92), <0.001	0.88 (0.83–0.94), <0.001
Gender			
Male	–	*REF*^c^	*REF*
Female	–	0.84 (0.79–0.90), <0.001	0.93 (0.87–0.99), 0.02
Age	–	1.12 (1.12–1.13), <0.001	1.11 (1.11–1.12), <0.001
Ethnicity			
White	–	*REF*	*REF*
Mixed	–	0.87 (0.55–1.39), 0.57	0.91 (0.57–1.45), 0.70
Asian	–	0.91 (0.72–1.17), 0.48	0.94 (0.74–1.21), 0.65
Black	–	1.25 (0.95–1.66), 0.11	1.26 (0.95–1.67), 0.11
Chinese	–	0.80 (0.20–3.18), 0.75	0.83 (0.21–3.31), 0.79
Other	–	0.78 (0.48–1.27), 0.32	0.81 (0.49–1.32), 0.39
Missing		0.97 (0.88–1.07), 0.54	0.97 (0.88–1.08), 0.61
IMD Decile	–	0.98 (0.97–0.99), <0.001	0.99 (0.98–0.999), 0.03
Number of therapy sessions attended	–	–	0.98 (0.98–0.99), <0.001
Psychotropic medications			
No	–	–	*REF*
Yes	–	–	1.17 (1.09–1.24), <0.001
Missing	–	–	1.14 (1.03–1.26), 0.01
Cardiovascular disease			
No	–	–	*REF*
Yes	–	–	2.00 (1.87–2.14), <0.001
Comorbid anxiety			
No	–	–	*REF*
Yes	–	–	0.89 (0.82–0.96), 0.004
Baseline depression severity	–	–	1.00 (0.998–1.01), 0.17
Long-term health condition			
No	–	–	*REF*
Yes	–	–	0.99 (0.93–1.06), 0.83
Missing	–	–	0.98 (0.90–1.07), 0.65

a Proportional hazards assumption: χ^2^(18) = 22.28, *p* = .22.

b Results are presented as: HR (95% CIs), *p*.

c Reference category.

The proportional hazards assumption was not violated (χ^2^ = 22.28, df = 18, *p* = 0.22). Findings from Models 1 (unadjusted) and 2 (adjusted for demographic covariates) were consistent with the above: reliable improvement in depressive symptoms was associated with reduced incidence of dementia diagnosis (Model 1: HR = 0.85, 95% CI 0.80–0.90, *p* < 0.001; Model 2: HR = 0.86, 95% CI 0.81–0.92, *p* < 0.001) (Table 3).

Models were run using the two commonest forms of dementia as the primary outcomes. Results showed that reliable improvement in depression was not significantly associated with reduced incidence of Alzheimer's disease, after full adjustment for all demographic and clinical covariates (HR = 0.91, 95% CI 0.83–1.00, *p* = 0.06). By contrast, there was a significant effect of reliable improvement in the depression on the incidence of vascular dementia (HR = 0.86, 95% CI 0.77–0.97, *p* = 0.01) (Table 4).

**Table 4.** Cox proportional hazards models to test associations between reliable improvement in depression following psychological therapy and dementia sub-types (Alzheimer's disease and vascular dementia)

	Alzheimer's disease *N* = 99 977	Vascular dementia *N* = 99 344
Reliable improvement in depression	0.91 (0.83–1.00), 0.06^a^	0.86 (0.77–0.97), 0.01
Gender	1.06 (0.96–1.17), 0.28	0.89 (0.71–0.90), <0.001
Age	1.12 (1.11–1.13), <0.001	1.12 (1.11–1.13), <0.001
Ethnicity		
White	*REF*	*REF*
Mixed	1.31 (0.72–2.37), 0.38	0.90 (0.37–2.17), 0.81
Asian	1.03 (0.71–1.49), 0.87	0.79 (0.48–1.29), 0.35
Black	1.14 (0.72–1.79), 0.58	1.50 (0.92–2.42), 0.10
Chinese	0.91 (0.13–6.46), 0.92	-
Other	0.96 (0.48–1.92), 0.91	0.34 (0.09–1.37), 0.13
Missing	1.01 (0.86–1.18), 0.94	0.96 (0.79–1.16), 0.65
Deprivation	0.99 (0.97–1.01), 0.24	0.97 (0.95–0.99), 0.001
Number of attended contacts	0.99 (0.97–0.999), 0.03	0.97 (0.95–0.99), <0.001
Taking psychotropic medications		
No	*REF*	*REF*
Yes	1.09 (0.98–1.20), 0.10	1.24 (1.10–1.40), <0.001
Missing	1.06 (0.91–1.24), 0.45	1.10 (0.90–1.33), 0.36
Cardiovascular disease		
No	*REF*	*REF*
Yes	1.48 (1.34–1.63), <0.001	4.07 (3.51–4.71), <0.001
Comorbid anxiety		
No	*REF*	*REF*
Yes	0.79 (0.70–0.89), <0.001	0.82 (0.70–0.95), 0.009
Baseline depression severity	1.00 (0.99–1.01), 0.56	1.01 (1.00–1.03), 0.03
Long-term health condition		
No	*REF*	*REF*
Yes	0.86 (0.78–0.96), 0.007	0.99 (0.87–1.13), 0.86
Missing	0.96 (0.85–1.08), 0.47	0.96 (0.82–1.12), 0.58

a Results are presented as HR (95% CIs), *p*.

### Supplementary analyses

Online Supplementary analyses including reliable recovery from depression and the continuous depression change score as the main predictors of dementia diagnosis were consistent with main analyses (Reliable recovery: HR = 0.87, 95% CI 0.82–0.92, *p* < 0.001; Continuous change: HR = 0.98, 95% CI 0.98–0.99, *p* < 0.001) (online Supplementary Tables S2 and S3). Models were re-run excluding cases with dementia diagnosed within 2 years of the psychological therapy to minimize the effects of any undiagnosed dementia present during therapy for depression. Results were consistent with those reported in the main analyses (HR = 0.92, 95% CI 0.85–0.99, *p* = 0.03), though the effect size was attenuated (online Supplementary Table S4). The model was re-run stratifying by median follow-up time (3.10 years). The model with shorter follow-up showed a significant effect of reliable improvement in the depression on dementia incidence (HR = 0.81, 95% CIs 0.74–0.88, *p* < 0.001). The model with the longer follow-up period did not show the same effect (HR = 0.95, 95% CI 0.87–1.05) *p* = 0.31) (online Supplementary Table S5). Models were run using only the sample with moderate or severe depressive symptoms at the start of treatment (⩾15 on the PHQ-9). The fully adjusted model showed that there was a significant association between reliable improvement in depressive symptoms following psychological therapy and dementia incidence (HR = 0.90, 95% CI 0.83–0.99, *p* *=* 0.03) (online Supplementary Table S6). Next, analyses were run excluding patients who reported taking psychotropic medications at the time of therapy. The fully adjusted model was consistent with the main models (HR = 0.90, 95% CI 0.83–0.99, *p* *=* 0.03) (online Supplementary Table S7). The fully adjusted model excluding participants who showed reliable deterioration in anxiety symptoms over therapy was also consistent with the main models (HR = 0.89, 95% CI 0.83–0.95, *p* < 0.001) (online Supplementary Table S8). The fully adjusted model including all-cause mortality as an event and dementia diagnosis as censored observations showed that reliable improvement in depression was associated with significantly reduced all-cause mortality (HR = 0.86, 95% CI 0.83–0.90), <0.001) (online Supplementary Table S9). Finally, an additional model was run including a random effect for the IAPT service that offered therapy to check if results were consistent after accounting for the potentially nested data structure. Results from these analyses were consistent with the main models (HR = 0.88, 95% CI 0.83–0.94, *p* < 0.001) (online Supplementary Table S10).

## Discussion

### Summary of main findings

Results from this study show that after adjustment for a range of demographic and clinical covariates, greater improvements in depression following psychological therapy are associated with reduced rates of dementia diagnosis in older people (age 65+). This finding remained consistent when alternative indicators of improvement in depressive symptoms were used (reliable recovery and continuous change scores), when excluding patients who showed reliable deterioration in anxiety over therapy, when accounting for variation within services, and when excluding patients who developed dementia within 2 years of ending psychological therapy (though the effect size was attenuated). In this sample of depressed older people, patients who achieved reliable improvement in depression across psychological therapy had a reduced rate of dementia diagnosis by about 12%, compared with those who did not achieve reliable improvement. Online Supplementary analyses showed that reliable improvement in depression was associated with reduced all-cause mortality, which suggests that the effects observed in main models are unlikely to be due to the competing risk of death. However, when stratifying by median follow-up time, the association was only significant in the shorter follow-up group.

It is important to note that this study does not address the question of causality between psychological therapy for depression and dementia incidence. Use of this study design can only test for associations. The processes underlying this association are unclear. Findings are consistent with at least four hypotheses, which are not mutually exclusive: (1) Psychological therapy to treat symptoms of depression may directly reduce risk or delay the onset of dementia, and may operate through various psychosocial [e.g. repetitive negative thinking (Marchant et al., 2020)], lifestyle [e.g. low levels of physical activity (Tan et al., 2017), etc.] and biological [e.g. cardiometabolic factors (John, Desai, Richards, Gaysina, & Stott, 2021; Newman et al., 2005), etc.] pathways. This is plausible because research has shown that depression may increase the risk for dementia [reviewed in 33] or alternatively may act as an accelerating factor which contributes towards conversion to and progression of dementia (Dafsari & Jessen, 2020). Psychological therapy to improve depression may therefore contribute towards reducing the risk of dementia or decelerating clinical conversion and progression of the disease. (2) Observed associations may be due to residual confounding by other causes (e.g. vascular pathology). (3) Neurodegeneration prior to the emergence of cognitive symptoms may have an effect on response to psychological therapies. As such, though cognitive deficits may not yet be clinically observable, people with a dementia prodrome may be less likely to recover in psychological therapies than those without or they may be more likely to drop out of therapy (reverse causality). This is plausible, given that effects of treatment outcome on dementia incidence did not hold for longer follow-up times, though it is unclear whether this is to do with the lower power of these analyses due to reduced numbers of dementia cases available in the sample.

It is notable that a high proportion of people in the sample were prescribed or taking psychotropic medications for their depressive symptoms. This is important, because in this study and in line with previous studies (Wang et al., 2018), taking psychotropic medications was associated with increased incidence of dementia in older people. The reason for this cannot be determined from this study. It is possible that psychotropic medications may be associated with dementia incidence through specific biological pathways, or this may be related to indication bias (Wang et al., 2016, 2018).

In addition, findings revealed that there was a small but significant association between length of treatment (i.e. number of sessions attended) and dementia incidence. This effect may be driven by number of mechanisms. Specifically, one possibility is that people who engage in therapy for longer may derive the most benefit in reducing symptoms of depression, which may be associated with reduced dementia incidence. It is also possible that groups who may be at increased risk of dementia (e.g. people who have chronic physical health conditions, or people from lower socioeconomic backgrounds, etc.) may be more likely to attend fewer sessions in IAPT. To minimize this possibility, the model was adjusted for a range of covariates, including the presence of long-term health conditions, and cardiovascular disease.

It is important to note that significant effects of reliable improvement in depression were observed on vascular dementia, and not Alzheimer's disease. This finding is in line with the literature which shows the strongest effects of depression on vascular dementia, compared with Alzheimer's disease (Diniz et al., 2013). In addition, some evidence has suggested that depression in later life may be part of an Alzheimer's disease prodrome, whereas recurrent depression may act as a risk factor for incident vascular dementia (Barnes et al., 2012). This may explain in part why improvements in depression were associated with the incidence of vascular dementia, but not Alzheimer's disease in this study.

As expected, greater age was associated with higher incidence of dementia. This is in line with evidence showing higher risk of dementia with greater age (McCullagh, Craig, McIlroy, & Passmore, 2001). Baseline level of depressive symptoms was not associated with dementia incidence. This is somewhat counter to work suggesting that higher levels of depression are associated with future dementia incidence (Da Silva et al., 2013).

### Strengths and limitations

Key strengths of this study are: (1) the availability of high-quality psychological therapy outcomes from all services in every health authority area of England; (2) the large sample size available for analysis (*N* = 119 808); and (3) the availability of variables known to be important both for depression treatment outcomes and potential dementia risks (e.g. deprivation), allowing for adjustment of these factors in analyses.

However, there are also several limitations. Firstly, there was no available measure of baseline cognition. It is therefore possible that lack of recovery in psychological therapy may be a marker for dementia prodrome. Though including baseline cognition would not fully address this issue, as cognitive problems may have not yet emerged. In addition, it is possible that participants may have been diagnosed with Mild Cognitive Impairment (MCI) prior to or during therapy, which may have affected therapy progress and outcomes. We attempted to partially account for these issues by excluding people who developed dementia within 1 year (and within 2 years in online Supplementary analyses) of psychological therapy, as it is possible these people may have been already experiencing cognitive impairments during their treatment.

Additionally, analyses were limited by the data available in routinely collected healthcare records, meaning there may be confounding factors unaccounted for. For example, vascular effects may be an important confounder in this association. To account for this, we adjusted for cardiovascular disease, though this may not completely address the issue. Data were not available on other important variables, including genetic risk, smoking, alcohol use, or family history of dementia, so residual confounding cannot be ruled out.

This study included a select sample of older people who have sought psychological treatment for their symptoms of depression (Saunders et al., 2021). It is estimated that 85% of older people with symptoms of depression do not get treatment from the NHS. Older people are five times less likely to access psychological therapies than young people in the UK, but six times more likely to be prescribed anti-depressant medication (Burns, 2015). Therefore, this sample of older people who did access psychological therapy may not be representative of the population of older people nationally. The study also could not account for other types of treatment offered after psychological therapy. For example, patients who did not respond to psychological therapies may be more likely to be offered antidepressant medication after therapy, compared to those whose symptoms improved. Additionally, there were no checks on the fidelity of treatment offered.

In addition, the majority of the sample was white with only 4.32% from ethnic minority backgrounds. It is therefore unclear whether results are generalizable to the wider population. It is important for future research to focus on the effects of psychological therapies on dementia incidence within under-represented groups, including people from ethnic minority backgrounds and people with different access to healthcare than those in the UK.

Misclassification of the outcome is plausible through several potential pathways: (1) Dementia diagnosis may not be recorded in the secondary datasets we have access to. We did not have access to primary care records, meaning some dementia diagnoses may have been missed, which may have biased results towards the null. However, the rate of misclassification as a result of this is likely to be low, because research has shown that there is high agreement rate in dementia case identification between HES data and GP records (Brown et al., 2016). (2) Data were only available for people with formally diagnosed dementia. It is known that many people experiencing symptoms of dementia may not have a formal diagnosis. As such, these individuals may have been missed in our analyses, potentially leading to misclassification and possibly attenuating effects. However, rates of dementia in this sample (4.46%) were in line with expected rates in a general population based on national prevalence estimates (Office for National Statistics, 2020).

Though people who had not yet been discharged from IAPT services were not specifically excluded from analyses, only six people meeting these criteria were included in the main models. It is therefore unlikely this exerted any disproportionate effects on results.

### Implications and future research

Evidence-based psychological therapies are a key treatment option for older people with depression. Although evidence shows that older people have better treatment outcomes from such therapies compared with younger people (Saunders et al., 2021), older people are underrepresented in these services, particularly those from ethnic minority backgrounds (Stickland & Gentry, 2016). Findings from this study show that beyond improving depression outcomes, psychological therapies may have additional benefits in reducing dementia incidence. Improving access for older people to evidence-based psychological therapies may help to maximize this benefit.

The processes underlying the association between psychological therapy outcomes and dementia incidence are not clear from this study. Future research should aim to distinguish between these possibilities, for example by including cognitive function at baseline, or testing over a longer follow-up period (ideally over several decades) with more cases. Testing whether psychological therapy to improve symptoms of depression in people already living with dementia is associated with slower cognitive decline would also be informative. Additionally, it may be important to investigate whether it is beneficial to incorporate psychological therapy for depression into future multi-domain prevention interventions for dementia. Finally, data on treatment outcomes for depression and anxiety (PHQ-9 and GAD-7) are critical in answering questions related to successful mental health treatment outcomes and the incidence of future health-related conditions. As such, it is important for other sectors of mental health services across the country to also collect and report outcome data and collate it nationally, ensuring questions like these can be answered more rigorously in the future.

### Conclusions

This study shows that effective treatment of depression through psychological therapy is associated with reduced rates of dementia diagnosis in older people (age 65+). Specifically, patients who achieved reliable improvement in depression symptoms across psychological therapy had a reduced risk of dementia diagnosis at any given point in time by about 12%, compared with those who did not achieve reliable improvement. These findings suggest that treating older people for depression through evidence-based psychological therapies may have additional benefits in reducing dementia incidence, as well as improving symptoms of depression.

## Supplementary material

For supplementary material accompanying this paper visit https://doi.org/10.1017/S0033291722002537. click here to view supplementary material
