# Supplementary material for: Associations between psychological therapy outcomes for depression and incidence of dementia
Source: Psychol Med. 2022 Sep 15;53(11):4869–79. doi: 10.1017/S0033291722002537 (PMC10476047; doi:10.1017/S0033291722002537)
Supplement: Supplementary file 1 [file S0033291722002537sup001.docx]

**Supplementary Materials**

**Supplementary Materials**

The Hospital Episode Statistics (HES) database contains information on all hospital admissions and outpatient appointments at National Health Service (NHS) hospitals in England. HES data are generated by patient administration systems within hospitals and then collected and processed by NHS Digital. HES records contain information about individual patients, including clinical variables (e.g. diagnoses, treatments, and operations), patient demographics (e.g. age, ethnicity, and gender), administrative information (e.g. admission and discharge times), and geographical information (e.g. area of residence, and area in which treatment was received).

The HES-ONS linked mortality data is generated through linkage of patient information from HES to mortality data from the Office of National Statistics (ONS). This dataset includes mortality information (including cause of and place of death) for people who have been treated in hospitals based in England (regardless of whether they died in hospital or not).

The Mental Health Services Data Set (MHSDS) (previously known as Mental Health Minimum Data Set – MHMDS, and as Mental Health and Learning Disabilities Data Set - MHLDDS) includes record-level information on secondary care services offered for people with mental illness, learning disability, and autism spectrum disorder. This includes services provided in hospitals, outpatient clinics, and in the community.

**List of Supplementary Tables**

**Supplementary Table 1:** Comparison between sample with missing data (N=46,609) and sample with complete data (N=73,199).

**Supplementary Table 2:** Cox proportional hazards models to test associations between reliable recovery from depression and dementia incidence.

**Supplementary Table 3:** Cox proportional hazards models to test associations between continuous depression change score and dementia incidence.

**Supplementary Table 4:** Cox proportional hazards models to test associations between reliable improvement from depression and dementia incidence, excluding dementia cases diagnosed within 2 years of IAPT attendance.

**Supplementary Table 5:** Cox proportional hazards models stratified by median follow up time.

**Supplementary Table 6:** Cox proportional hazards models to test associations between reliable improvement from depression and dementia incidence, including only the sample with moderate or severe depressive symptoms.

**Supplementary Table 7:** Cox proportional hazards models to test associations between reliable improvement from depression and dementia incidence, excluding patients who reported taking psychotropic medications.

**Supplementary Table 8:** Cox proportional hazards models to test associations between reliable improvement from depression and dementia incidence, excluding participants who showed reliable deterioration in anxiety symptoms.

**Supplementary Table 9:** Cox proportional hazards models including all-cause mortality as an event and dementia diagnosis as censored observations.

**Supplementary Table 10:** Model including random effect of IAPT service.

**Supplementary Table 1: Comparison between sample with missing data (N=46,609) and sample with complete data (N=73,199).**

**Odel**

|  | **Sample with missing data (N=46,609)** | **Sample with complete data (N=73,199)** | **T-test/Chi-square** |
| --- | --- | --- | --- |
| Reliable improvement in depression: N (%) |  |  |  |
| No | 12,434 (37.83) | 24,648 (33.67) | χ2(1)=172.25, p<.001 |
| Yes | 20,436 (62.17) | 48,551 (66.33) |  |
| Reliable recovery in depression: N (%) |  |  |  |
| No | 14,799 (45.24) | 29,855 (40.79) | χ2(1)=183.83, p<.001 |
| Yes | 17,914 (54.76) | 43,344 (59.21) |  |
| Continuous change in depression score: Mean (SD) | 6.17 (6.85) | 7.85 (6.69) | t(119391)=-41.72, p<.001 |
| Gender: N (%) |  |  |  |
| Male | 14,477 (31.36) | 23,794 (32.51) | χ2(1)=16.92, p<.001 |
| Female | 31,680 (68.64) | 49,405 (67.49) |  |
| Age: Mean (SD) | 71.60 (5.82) | 71.52 (5.64) | t(119806)=2.26, p=.02 |
| Ethnicity: N (%) |  |  |  |
| White | 33,581 (95.33) | 70,156 (95.84) | χ2(5)=34.95, p<.001 |
| Mixed | 242 (0.69) | 335 (0.46) |  |
| Asian | 776 (2.20) | 1,554 (2.12) |  |
| Black | 341 (0.97) | 681 (0.93) |  |
| Chinese | 35 (0.10) | 51 (0.07) |  |
| Other | 251 (0.71) | 422 (0.58) |  |
| IMD Decile: Mean (SD) | 5.76 (2.81) | 5.99 (2.74) | t(115807)=-14.13, p<.001 |
| Number of therapy sessions attended: Mean (SD) | 5.88 (4.09) | 6.45 (4.13) | t(119806)=-23.28, p<.001 |
| Psychotropic medications: N (%) |  |  |  |
| No | 14,662 (43.74) | 32,327 (44.16) | χ2(1)=1.66, p=.20 |
| Yes | 18,858 (56.26) | 40,872 (55.84) |  |
| Cardiovascular disease: N (%) |  |  |  |
| No | 25,895 (55.67) | 42,420 (57.95) | χ2(1)=60.48, p<.001 |
| Yes | 20,621 (44.33) | 30,779 (42.05) |  |
| Comorbid anxiety: N (%) |  |  |  |
| No | 6,345 (13.72) | 9,690 (13.24) | χ2(1)=5.69, p=.02 |
| Yes | 39,898 (86.28) | 63,509 (86.76) |  |
| Baseline depression severity: Mean (SD) | 15.69 (4.67) | 15.80 (4.54) | t(119502)=-4.08, p<.001 |
| Long-term health condition: N (%) |  |  |  |
| No | 8,793 (44.90) | 32,509 (44.41) | χ2(1)=1.48, p=.22 |
| Yes | 10,791 (55.10) | 40,690 (55.59) |  |

**Supplementary Table 2: Cox proportional hazards models to test associations between reliable recovery from depression and dementia incidence.**

|  | **Model 1  (Unadjusted)** | **Model 2  (Adjusted for demographic covariates)** | **Model 3  (Adjusted for demographic and clinical covariates)** |
| --- | --- | --- | --- |
| Reliable recovery from depression | 0.85 (0.80-0.90), <.001* | 0.85 (0.80-0.90), <.001 | 0.87 (0.82-0.92), <.001 |
| Gender |  |  |  |
| Male | - | *REF* | *REF* |
| Female | - | 0.84 (0.79-0.90), <.001 | 0.93 (0.87-0.99), .02 |
| Age | - | 1.13 (1.12-1.13), <.001 | 1.11 (1.11-1.12), <.001 |
| Ethnicity |  |  |  |
| White | - | *REF* | *REF* |
| Mixed | - | 0.87 (0.55-1.39), 57 | 0.91 (0.57-1.45), .70 |
| Asian | - | 0.91 (0.72-1.17), .48 | 0.94 (0.74-1.21), .65 |
| Black | - | 1.22 (0.92-1.62), .16 | 1.26 (0.95-1.67), .11 |
| Chinese | - | 0.79 (0.20-3.15), .74 | 0.82 (0.20-3.28), .78 |
| Other | - | 0.78 (0.48-1.27), .32 | 0.80 (0.49-1.31), .38 |
| Missing |  | 0.97 (0.88-1.07), .57 | 0.97 (0.88-1.08), .60 |
| IMD Decile | - | 0.98 (0.97-0.99), <.001 | 0.99 (0.98-0.999), .03 |
| Number of IAPT sessions attended | - | - | 0.98 (0.98-0.99), <.001 |
| Psychotropic medication use |  |  |  |
| No | - | - | *REF* |
| Yes | - | - | 1.17 (1.09-1.24), <.001 |
| Missing | - | - | 1.14 (1.03-1.26), .01 |
| Cardiovascular disease |  |  |  |
| No | - | - | *REF* |
| Yes | - | - | 2.00 (1.87-2.14), <.001 |
| Comorbid anxiety |  |  |  |
| No | - | - | *REF* |
| Yes | - | - | 0.89 (0.82-0.96), .004 |
| Baseline depression severity | - | - | 1.002 (0.996-1.01), .50 |
| Long-term health condition |  |  |  |
| No | - | - | *REF* |
| Yes | - | - | 0.99 (0.92-1.06), .82 |
| Missing | - | - | 0.98 (0.90-1.06), .64 |

* Results are presented as: HR (95% CI), p.

**Supplementary Table 3: Cox proportional hazards models to test associations between continuous depression change score and dementia incidence.**

|  | **Model 1  (Unadjusted)** | **Model 2  (Adjusted for demographic covariates)** | **Model 3  (Adjusted for demographic and clinical covariates)** |
| --- | --- | --- | --- |
| Continuous change in depression | 0.98 (0.98-0.98), <.001* | 0.98 (0.98-0.99), <.001 | 0.98 (0.98-0.99), <.001 |
| Gender |  |  |  |
| Male | - | *REF* | *REF* |
| Female | - | 0.83 (0.78-0.88), <.001 | 0.92 (0.87-0.98), .007 |
| Age | - | 1.12 (1.11-1.12), <.001 | 1.11 (1.10-1.11), <.001 |
| Ethnicity |  |  |  |
| White | - | *REF* | *REF* |
| Mixed | - | 0.84 (0.54-1.28), 41 | 0.88 (0.57-1.35), .55 |
| Asian | - | 0.95 (0.76-1.19), .65 | 0.98 (0.78-1.23), .87 |
| Black | - | 1.27 (0.98-1.64), .08 | 1.30 (1.00-1.69), .05 |
| Chinese | - | 0.82 (0.26-2.54), .73 | 0.90 (0.29-2.78), .85 |
| Other | - | 0.79 (0.50-1.24), .30 | 0.81 (0.51-1.27), .35 |
| Missing |  | 0.95 (0.87-1.04), .24 | 0.99 (0.90-1.08), .78 |
| IMD Decile | - | 0.98 (0.97-0.99), .002 | 0.99 (0.98-1.00), .10 |
| Number of IAPT sessions attended | - | - | 0.99 (0.98-0.99), <.001 |
| Psychotropic medication use |  |  |  |
| No | - | - | *REF* |
| Yes | - | - | 1.17 (1.10-1.24), <.001 |
| Missing | - | - | 1.11 (1.02-1.22), .02 |
| Cardiovascular disease |  |  |  |
| No | - | - | *REF* |
| Yes | - | - | 2.04 (1.92-2.17), <.001 |
| Comorbid anxiety |  |  |  |
| No | - | - | *REF* |
| Yes | - | - | 0.87 (0.81-0.94), .001 |
| Baseline depression severity | - | - | 1.02 (1.01-1.02), <.001 |
| Long-term health condition |  |  |  |
| No | - | - | *REF* |
| Yes | - | - | 0.98 (0.92-1.05), .60 |
| Missing | - | - | 0.97 (0.89-1.04), .38 |

* Results are presented as: HR (95% CI), p

**Supplementary Table 4: Cox proportional hazards models to test associations between reliable improvement from depression and dementia incidence, excluding dementia cases diagnosed within 2 years of IAPT attendance.**

|  | **Model 1  (Unadjusted)** | **Model 2  (Adjusted for demographic covariates)** | **Model 3  (Adjusted for demographic and clinical covariates)** |
| --- | --- | --- | --- |
| Reliable improvement from depression | 0.90 (0.83-0.96), .002* | 0.91 (0.84-0.97), .007 | 0.92 (0.85-0.99), .03 |
| Gender |  |  |  |
| Male | - | *REF* | *REF* |
| Female | - | 0.85 (0.79-0.91), <.001 | 0.93 (0.86-1.00), .06 |
| Age | - | 1.13 (1.12-1.13), <.001 | 1.12 (1.11-1.12), <.001 |
| Ethnicity |  |  |  |
| White | - | *REF* | *REF* |
| Mixed | - | 0.67 (0.36-1.25), 21 | 0.70 (0.38-1.31), .27 |
| Asian | - | 0.86 (0.64-1.17), .35 | 0.89 (0.66-1.22), .48 |
| Black | - | 1.42 (1.03-1.95), .03 | 1.41 (1.02-1.95), .04 |
| Chinese | - | 1.15 (0.29-4.59), .85 | 1.20 (0.30-4.79), .80 |
| Other | - | 0.87 (0.49-1.53), .63 | 0.91 (0.51-1.60), .73 |
| Missing |  | 1.02 (0.91-1.14), .73 | 1.04 (0.92-1.17), .56 |
| IMD Decile | - | 0.98 (0.97-0.99), .004 | 0.99 (0.98-1.00), .09 |
| Number of IAPT sessions attended | - | - | 0.99 (0.98-0.997), .009 |
| Psychotropic medication use |  |  |  |
| No | - | - | *REF* |
| Yes | - | - | 1.17 (1.08-1.26), <.001 |
| Missing | - | - | 1.13 (1.01-1.28), .04 |
| Cardiovascular disease |  |  |  |
| No | - | - | *REF* |
| Yes | - | - | 1.97 (1.82-2.13), <.001 |
| Comorbid anxiety |  |  |  |
| No | - | - | *REF* |
| Yes | - | - | 0.93 (0.84-1.03), .16 |
| Baseline depression severity | - | - | 1.00 (0.995-1.01), .41 |
| Long-term health condition |  |  |  |
| No | - | - | *REF* |
| Yes | - | - | 1.03 (0.94-1.12), .53 |
| Missing | - | - | 0.99 (0.90-1.09), .86 |

* Results are presented as: HR (95% CI), p

**Supplementary Table 5: Cox proportional hazards models stratified by median follow up time.**

|  | **Short follow up  (< 3.10 years)** | **Long follow up  (> 3.10 years)** |
| --- | --- | --- |
| Reliable improvement in depression | 0.81 (0.74-0.88), <.001* | 0.95 (0.87-1.05), .31 |
| Gender |  |  |
| Male | *REF* | *REF* |
| Female | 1.01 (0.92-1.09), .89 | 0.94 (0.85-1.03), .21 |
| Age | 1.09 (1.08-1.09), <.001 | 1.11 (1.11-1.12), <.001 |
| Ethnicity |  |  |
| White | *REF* | *REF* |
| Mixed | 1.16 (0.62-2.16), .64 | 0.88 (0.44-1.75), .71 |
| Asian | 0.96 (0.71-1.31), .81 | 0.81 (0.54-1.23), .33 |
| Black | 1.18 (0.82-1.71), .38 | 1.21 (0.78-1.88), .40 |
| Chinese | 0.58 (0.08-4.12), .59 | 1.13 (0.16-8.04), .90 |
| Other | 0.53 (0.26-1.06), .07 | 1.04 (0.52-2.09), .91 |
| Missing | 1.01 (0.87-1.16), .93 | 0.93 (0.79-1.08), .33 |
| IMD Decile | 0.99 (0.98-1.01), .34 | 0.99 (0.97-1.00), .14 |
| Number of therapy sessions attended | 0.98 (0.97-0.99), .001 | 0.98 (0.97-0.99), .004 |
| Taking psychotropic medications |  |  |
| No | *REF* | *REF* |
| Yes | 1.19 (1.09-1.30), <.001 | 1.17 (1.06-1.29),.002 |
| Missing | 1.63 (1.42-1.87), <.001 | 1.01 (0.87-1.18), .88 |
| Cardiovascular disease |  |  |
| No | *REF* | *REF* |
| Yes | 2.26 (2.07-2.47), <.001 | 2.10 (1.90-2.32), <.001 |
| Comorbid anxiety |  |  |
| No | *REF* | *REF* |
| Yes | 0.88 (0.79-0.98), .02 | 0.96 (0.84-1.09), .55 |
| Baseline depression severity | 1.00 (0.99-1.01), .97 | 1.00 (0.99-1.01), .60 |
| Long-term health condition |  |  |
| No | *REF* | *REF* |
| Yes | 0.82 (0.75-0.90), <.001 | 1.01 (0.90-1.12), .90 |
| Missing | 1.12 (0.996-1.26), .06 | 1.02 (0.91-1.16), .69 |

* Results are presented as: HR (95% CI), p

**Supplementary Table 6: Cox proportional hazards models to test associations between reliable improvement from depression and dementia incidence, including only the sample with moderate or severe depressive symptoms.**

|  | **Model 1  (Unadjusted)** | **Model 2  (Adjusted for demographic covariates)** | **Model 3  (Adjusted for demographic and clinical covariates)** |
| --- | --- | --- | --- |
| Reliable improvement from depression | 0.91 (0.84-0.998), .04* | 0.88 (0.80-0.96), .003 | 0.90 (0.83-0.99), .03 |
| Gender |  |  |  |
| Male | - | *REF* | *REF* |
| Female | - | 0.80 (0.74-0.88), <.001 | 0.89 (0.82-0.97), .01 |
| Age | - | 1.13 (1.12-1.13), <.001 | 1.12 (1.11-1.12), <.001 |
| Ethnicity |  |  |  |
| White | - | *REF* | *REF* |
| Mixed | - | 0.72 (0.37-1.38), .32 | 0.74 (0.39-1.43), .38 |
| Asian | - | 0.72 (0.51-1.03), .07 | 0.75 (0.53-1.08), .12 |
| Black | - | 1.30 (0.90-1.88), .16 | 1.31 (0.90-1.90), .16 |
| Chinese | - | 1.66 (0.41-6.63), .48 | 1.72 (0.43-6.89), .4 |
| Other | - | 0.83 (0.43-1.61), .59 | 0.85 (0.44-1.64), .63 |
| Missing |  | 0.96 (0.84-1.10), .57 | 0.97 (0.84-1.12), .70 |
| IMD Decile | - | 0.98 (0.97-0.997), .02 | 0.99 (0.98-1.01), .26 |
| Number of IAPT sessions attended | - | - | 0.98 (0.97-0.99), .003 |
| Psychotropic medication use |  |  |  |
| No | - | - | *REF* |
| Yes | - | - | 1.15 (1.05-1.25), .003 |
| Missing | - | - | 1.12 (0.97-1.29), .12 |
| Cardiovascular disease |  |  |  |
| No | - | - | *REF* |
| Yes | - | - | 2.14 (1.95-2.34), <.001 |
| Comorbid anxiety |  |  |  |
| No | - | - | *REF* |
| Yes | - | - | 0.84 (0.72-0.97), .02 |
| Baseline depression severity | - | - | 1.01 (0.997-1.02), .13 |
| Long-term health condition |  |  |  |
| No | - | - | *REF* |
| Yes | - | - | 1.00 (0.91-1.10), .98 |
| Missing | - | - | 1.00 (0.89-1.12), .998 |

* Results are presented as: HR (95% CI), p

**Supplementary Table 7: Cox proportional hazards models to test associations between reliable improvement from depression and dementia incidence, excluding patients who reported taking psychotropic medications.**

|  | **Model 1  (Unadjusted)** | **Model 2  (Adjusted for demographic covariates)** | **Model 3  (Adjusted for demographic and clinical covariates)** |
| --- | --- | --- | --- |
| Reliable improvement from depression | 0.87 (0.80-0.95), .001* | 0.89 (0.82-0.97), .008 | 0.90 (0.83-0.99), .03 |
| Gender |  |  |  |
| Male | - | *REF* | *REF* |
| Female | - | 0.80 (0.73-0.87), <.001 | 0.88 (0.80-0.96), .005 |
| Age | - | 1.13 (1.12-1.14), <.001 | 1.12 (1.11-1.12), <.001 |
| Ethnicity |  |  |  |
| White | - | *REF* | *REF* |
| Mixed | - | 0.57 (0.27-1.19), .14 | 0.58 (0.27-1.21), .15 |
| Asian | - | 0.87 (0.63-1.21), .41 | 0.88 (0.63-1.22), .44 |
| Black | - | 1.44 (1.03-2.02), .03 | 1.49 (1.06-2.08), .02 |
| Chinese | - | 1.41 (0.35-5.66), .62 | 1.40 (0.35-5.62), .63 |
| Other | - | 0.79 (0.42-1.47), .45 | 0.81 (0.44-1.52), .52 |
| Missing |  | 1.01 (0.89-1.16), .84 | 1.01 (0.87-1.16), .93 |
| IMD Decile | - | 0.98 (0.97-0.996), .02 | 0.99 (0.97-1.00), .10 |
| Number of IAPT sessions attended | - | - | 0.98 (0.97-0.99), .002 |
| Cardiovascular disease |  |  |  |
| No | - | - | *REF* |
| Yes | - | - | 1.93 (1.75-2.12), <.001 |
| Comorbid anxiety |  |  |  |
| No | - | - | *REF* |
| Yes | - | - | 0.90 (0.80-1.01), .08 |
| Baseline depression severity | - | - | 1.01 (0.997-1.02), .15 |
| Long-term health condition |  |  |  |
| No | - | - | *REF* |
| Yes | - | - | 0.94 (0.85-1.05), .28 |
| Missing | - | - | 0.96 (0.85-1.08), .47 |

* Results are presented as: HR (95% CI), p

**Supplementary Table 8:** **Cox proportional hazards models to test associations between reliable improvement from depression and dementia incidence, excluding participants who showed reliable deterioration in anxiety symptoms.**

|  | **Model 1  (Unadjusted)** | **Model 2  (Adjusted for demographic covariates)** | **Model 3  (Adjusted for demographic and clinical covariates)** |
| --- | --- | --- | --- |
| Reliable improvement from depression | 0.85 (0.80-0.91), <.001 | 0.87 (0.82-0.93), <.001 | 0.89 (0.83-0.95), <.001 |
| Gender |  |  |  |
| Male | - | *REF* | *REF* |
| Female | - | 0.84 (0.79-0.90), <.001 | 0.92 (0.87-0.99), .02 |
| Age | - | 1.13 (1.12-1.13), <.001 | 1.11 (1.11-1.12), <.001 |
| Ethnicity |  |  |  |
| White | - | *REF* | *REF* |
| Mixed | - | 0.79 (0.48-1.31), .36 | 0.83 (0.50-1.39), .48 |
| Asian | - | 0.91 (0.70-1.18), .49 | 0.95 (0.73-1.23), .69 |
| Black | - | 1.12 (0.81-1.53), .50 | 1.13 (0.82-1.56), .44 |
| Chinese | - | 0.86 (0.22-3.44), .83 | 0.91 (0.23-3.64), .89 |
| Other | - | 0.70 (0.41-1.21), .21 | 0.74 (0.43-1.27), .27 |
| Missing |  | 0.97 (0.88-1.07), .55 | 0.97 (0.87-1.08), .59 |
| IMD Decile | - | 0.98 (0.97-0.99), .002 | 0.99 (0.98-1.00), .09 |
| Number of IAPT sessions attended | - | - | 0.98 (0.97-0.99), <.001 |
| Psychotropic medication use |  |  |  |
| No | - | - | *REF* |
| Yes | - | - | 1.17 (1.09-1.25), <.001 |
| Missing | - | - | 1.15 (1.03-1.27), .01 |
| Cardiovascular disease |  |  |  |
| No | - | - | *REF* |
| Yes | - | - | 2.01 (1.87-2.15), <.001 |
| Comorbid anxiety |  |  |  |
| No | - | - | *REF* |
| Yes | - | - | 0.90 (0.82-0.99), .02 |
| Baseline depression severity | - | - | 1.00 (0.997-1.01), .25 |
| Long-term health condition |  |  |  |
| No | - | - | *REF* |
| Yes | - | - | 0.98 (0.91-1.05), .54 |
| Missing | - | - | 0.98 (0.90-1.07), .67 |

* Results are presented as: HR (95% CI), p

**Supplementary Table 9:** **Cox proportional hazards models including all-cause mortality as an event and dementia diagnosis as censored observations.**

|  | **Model 1  (Unadjusted)** | **Model 2  (Adjusted for demographic covariates)** | **Model 3  (Adjusted for demographic and clinical covariates)** |
| --- | --- | --- | --- |
| Reliable improvement from depression | 0.84 (0.81-0.88), <.001* | 0.86 (0.82-0.89), <.001 | 0.86 (0.83-0.90), <.001 |
| Gender |  |  |  |
| Male | - | *REF* | *REF* |
| Female | - | 0.60 (0.58-0.62), <.001 | 0.69 (0.66-0.72), <.001 |
| Age | - | 1.11 (1.10-1.11), <.001 | 1.09 (1.09-1.09), <.001 |
| Ethnicity |  |  |  |
| White | - | *REF* | *REF* |
| Mixed | - | 0.85 (0.62-1.16), .30 | 0.87 (0.63-1.18), .36 |
| Asian | - | 0.66 (0.55-0.80), <.001 | 0.66 (0.55-0.79), <.001 |
| Black | - | 0.60 (0.46-0.79), <.001 | 0.61 (0.47-0.80), <.001 |
| Chinese | - | 1.24 (0.59-2.61), .56 | 1.30 (0.62-2.72), .49 |
| Other | - | 0.72 (0.51-1.00), .05 | 0.72 (0.52-1.00), .05 |
| Missing |  | 0.96 (0.90-1.02), .17 | 1.03 (0.96-1.10), .44 |
| IMD Decile | - | 0.95 (0.95-0.96), <.001 | 0.97 (0.96-0.97), <.001 |
| Number of IAPT sessions attended | - | - | 0.99 (0.98-0.99), <.001 |
| Psychotropic medication use |  |  |  |
| No | - | - | *REF* |
| Yes | - | - | 1.09 (1.05-1.14), <.001 |
| Missing | - | - | 1.18 (1.10-1.26), <.001 |
| Cardiovascular disease |  |  |  |
| No | - | - | *REF* |
| Yes | - | - | 2.62 (2.50-2.74), <.001 |
| Comorbid anxiety |  |  |  |
| No | - | - | *REF* |
| Yes | - | - | 0.86 (0.82-0.91), <.001 |
| Baseline depression severity | - | - | 1.02 (1.01-1.02), <.001 |
| Long-term health condition |  |  |  |
| No | - | - | *REF* |
| Yes | - | - | 1.38 (1.32-1.45), <.001 |
| Missing | - | - | 1.06 (1.00-1.12), .05 |

* Results are presented as: HR (95% CI), p

**Supplementary Table 10: Model including random effect of IAPT service.**

|  | **Model 1  (Unadjusted)** | **Model 2  (Adjusted for demographic covariates)** | **Model 3  (Adjusted for demographic and clinical covariates)** |
| --- | --- | --- | --- |
| Reliable improvement from depression | 0.85 (0.80-0.90), <.001* | 0.86 (0.81-0.92), <.001 | 0.88 (0.83-0.94), <.001 |
| Gender |  |  |  |
| Male | - | *REF* | *REF* |
| Female | - | 0.84 (0.79-0.89), <.001 | 0.92 (0.87-0.98), .01 |
| Age | - | 1.13 (1.12-1.13), <.001 | 1.11 (1.11-1.12), <.001 |
| Ethnicity |  |  |  |
| White | - | *REF* | *REF* |
| Mixed | - | 0.85 (0.53-1.35), 49 | 0.89 (0.56-1.41), .61 |
| Asian | - | 0.90 (0.70-1.15), .40 | 0.92 (0.72-1.18), .52 |
| Black | - | 1.23 (0.92-1.63), .16 | 1.22 (0.92-1.63), .17 |
| Chinese | - | 0.84 (0.21-3.36), .81 | 0.86 (0.21-3.45), .83 |
| Other | - | 0.78 (0.48-1.28), .33 | 0.80 (0.49-1.31), .38 |
| Missing |  | 0.96 (0.87-1.06), .42 | 0.99 (0.89-1.10), .87 |
| IMD Decile | - | 0.98 (0.97-0.99), <.001 | 0.99 (0.97-0.997), .01 |
| Number of IAPT sessions attended | - | - | 0.98 (0.98-0.99), <.001 |
| Psychotropic medication use |  |  |  |
| No | - | - | *REF* |
| Yes | - | - | 1.17 (1.09-1.25), <.001 |
| Missing | - | - | 1.13 (1.02-1.26), .02 |
| Cardiovascular disease |  |  |  |
| No | - | - | *REF* |
| Yes | - | - | 1.99 (1.86-2.12), <.001 |
| Comorbid anxiety |  |  |  |
| No | - | - | *REF* |
| Yes | - | - | 0.88 (0.81-0.96), .003 |
| Baseline depression severity | - | - | 1.00 (0.998-1.01), .19 |
| Long-term health condition |  |  |  |
| No | - | - | *REF* |
| Yes | - | - | 1.01 (0.94-1.08), .86 |
| Missing | - | - | 0.93 (0.85-1.01), .08 |

* Results are presented as: HR (95% CI), p
